# Skin microbiome considerations for long haul space flights

**DOI:** 10.3389/fcell.2022.956432

**Published:** 2022-09-08

**Authors:** Gabrielle Caswell, Ben Eshelby

**Affiliations:** ^1^ Spaceport Australia, Moree, NSW, Australia; ^2^ Formulae, Albury, NSW, Australia

**Keywords:** space flight, skin pH, microgravity, 3D molecular structure, immuno-suppression, Vitamin D, microbiome, dysbiosis

## Abstract

Dysbiosis of the human skin microbiome has long been associated with changes to the pH of the skin, dermal immune function and chronic skin conditions. Dermatological issues have been noted as the most prevalent medical presentation in the microgravity environment of space. The change in gravitational forces has been implicated in human immuno-suppression, also impacted by changes in the gastrointestinal-skin axis and its impact on Vitamin D metabolism, altered microbial gene expression in resident flora (leading changes in biofilm formation) and increased virulence factors in potential pathogens. There are also other stressors to the skin microbiome unique to space travel, including increased exposure to radiation, prolonged periods of dry washing technique, air quality and changes in microbe replication and growth parameters. Optimal microbiome health leads to enhanced skin barrier manufacture and maintenance, along with improved skin immune function and healing. In a microgravity environment expected to be experienced during long space flights, disruptions to the skin microbiome, coupled with increased virulence of pathological viruses and bacteria has implications for holistic skin health, astronaut cognitive function and mental health, and is coupled with slowed rates of wound healing. Scenario management for holistic skin health and restoration of microbiome homeostasis on long space flights require consideration.

## Introduction

Our skin microbiome has evolved, and continues to evolve on Earth as a commensal system, vital for the health and life of the individual. The challenges of space flight on human health and physiology is a relatively new area of research, and as humankind’s ambition to move towards space colonization increases, there is increased likelihood of new disease encounters and physiological dysfunction of astronauts skin (Taylor, 1993; Horneck et al., 2003; Horneck and Comet, 2006; Taylor, 2015). Identifying methods to preserve the health of the commensal organisms that make up the human skin microbiome, and thus the overall health of the skin, during long haul space flight is of great clinical importance.

Thus far, human microgravity experience is limited to missions on the International Space Station (ISS) and Salyut, and Skylab stations, with additional information gained from space shuttle data collections and isolated analog experiments. There are numerous factors at play which affect the skin differently to that on Earth, skin microbiome changes are noted and further complicated by differences in microbial growth rates in microgravity (Taylor, 2015). Clinicians have been cognisant of medically important microorganisms populating astronauts early in the history of space flight (Coiletti Louis et al., 1991; Taylor, 1993; Williams, 2003a; Taylor, 2015). Changes to the human immune system, even after short-duration space flights, have been noted (Taylor, 1993). Physiological processes in the skin contribute to wound heating, multi-system health, mental health and cognitive functioning and is a relatively neglected area of space medicine.

## Microgravity, immunosupression, and Vitamin D production

For reasons not yet ascertained, microgravity environments affect the human immune response; this effect has been recorded after relatively low exposure (Nefedov et al., 1971; Stowe et al., 2001; Williams, 2003a; Mehta et al., 2014). Some of the catalogued changes to the human immune system range from differences in cellular genetic expression, changes to specific classes of immune cell activation (i.e., monocytes, leukocytes), interruptions of the gastrointestinal-skin axis, changes in cellular response (i.e., monocyte cellular imbalance) to endotoxins (cytokine blunting), as well as changes in the growth and nature of common commensals, pathogenic bacteria and viruses (Canova Sabrina et al., 2005; Kaur et al., 2005; Kaur et al., 2008; Baqai et al., 2009; Guéguinou et al., 2009; McPhee and Charles, 2010; Jennifer et al., 2021; Spatz et al., 2021). It is hypothesised, but the mechanism not fully understood, physiological changes monitored in humans may be directly influenced by the microgravity environment of space. With a resulting change of tertiary and quaternary molecular conformation leading, for example, to molecular conformational mismatch and structural stress, in turn causing cytokine blunting, affecting the inflammatory mediated immune response with dysregulation of signaling and recruitment pathways (Nickerson et al., 2000; Kaur et al., 2005; Kaur et al., 2008; Guéguinou et al., 2009; Picardo and Monica, 2014; Jennifer et al., 2021; Spatz et al., 2021). Consideration should also be given to the formation of lymph and its propulsion within the human lymph system in microgravity environments. Lymph contributes to fluid homeostasis of the interstitial and serosal cavities, but also acts as an “immune cell highway” propelling classes of immune cells to sites of immune responses as part of the immune reaction (Canova Sabrina et al., 2005; Kaur et al., 2005; Kaur et al., 2008; Baqai et al., 2009; Guéguinou et al., 2009; McPhee and Charles, 2010; Jennifer et al., 2021; Solari et al., 2021; Spatz et al., 2021). It is noted ISS astronauts experience gross disturbance of this system (evidenced by pseudo-sinusitis and “puffy faces,” which do not respond to traditional diuretics) in microgravity (Solari et al., 2021).

Recent enclosed analog research has been focussed on the biological translocation of bacteria within closed environments/habitats, potential bacterial-derived toxicity, as well as exploration of tissue-derived toxins (involving the lymph system). However the contributing impact of microgravity on the lymphatic system has not been extensively explored (Zwart et al., 2011; SanMiguel and Grice, 2015; Cabalín et al., 2021; Tang et al., 2021). Solari et.al. (2021) note that there is little data on the modulation of gut lymphatic drainage and movement in disease management, and attention to lymph drainage remains the domain of specific areas of cancer management (i.e., breast cancer lymphoedema) (Smith et al., 1999; Zwart et al., 2011; SanMiguel and Grice, 2015; Lund et al., 2016; Schwager and Detmar, 2019; Cabalín et al., 2021; Solari et al., 2021). Limited research has reviewed the impact of artificially imposed lymph flow perhaps improving the composition of gut microbiome, which may in turn impact or reduce future gut-microbiome imbalance associated illnesses and nutritional deficiencies. A secondary effect may be the improved immunosurveillance and delivery of immune cells. (Smith et al., 2005; Scott et al., 2021; Smith et al., 2021; Solari et al., 2021; Tang et al., 2021).

Nutrient studies demonstrate that lipid transport is associated closely with the gut microenvironment; changes to this environment can affect the absorption of dietary lipids and their associated lipid soluble nutrients. In turn, changed gut microenvironments impact on the effective immunosurveillance between the villi interstitial space and mesenteric lymph nodes (Solari et al., 2021). It is appreciated that there are widespread associations: reduced lipid transport into the blood impacts immunosurveillance and increases risk tissue oedema (an independent risk factor for infection); and reduced dietary lipid transport affects uptake of vital nutrients and mineral co-enzymes ultimately affecting the gastrointestinal-skin axis. (Sonnenfield and Shearer, 2002; Smith et al., 2005; Zwart et al., 2011; Zwart et al., 2013; Carlson, 2014; Shen et al., 2014; SanMiguel and Grice, 2015; Anna et al., 2016; Scott et al., 2021; Smith et al., 2021; Solari et al., 2021; Tang et al., 2021).

More recently noted effects also attributed to microgravity affects include altered T cell activation, decreased sensitivity and activation of molecular signalling pathways (leading to reduced functionality of protein co-activators) by mechanisms yet to be fully elucidated (Nickerson et al., 2000; Sonnenfield and Shearer, 2002; Kaur et al., 2008; Zwart et al., 2011; Zwart et al., 2013; Carlson, 2014; Picardo and Monica, 2014; Shen et al., 2014; SanMiguel and Grice, 2015; Anna et al., 2016) A recent study confirmed the ability of T cells to influence the bacterial microbiome of the skin (Shen et al., 2014). An example, the reactivation of latent viral infections, due in part to the de-activation or down regulation of some parts of the human immune system, has been observed (Stowe et al., 2001; Baqai et al., 2009; Zwart et al., 2013; Carlson, 2014; Taylor, 2015). Further studies with regards to primary immuno-deficiencies show increased microbial permissiveness and growth in opportunistic pathogens (SanMiguel and Grice, 2015).

Vitamin D plays a cardinal role regulating the human skin’s immune response and contributes to the healing potential of the skin. Low Vitamin D status leads to increased risk of topical infections and systemic and multi-organ changes (i.e. osteoporosis, pulmonary fibrosis); also noted is decreased skin immune responses with disordered wound healing (Smith et al., 1999; Zwart et al., 2011; Schwager and Detmar, 2019; Cabalín et al., 2021; Smith et al., 2021; Tang et al., 2021). The immunological mechanisms required for the production of Vitamin D via the 7-DHC pathway can be clinically interrupted in enclosed space environments. The regulation of keratinocyte differentiation is dependent on Vitamin D, creating a nexus between the body’s Vitamin D stocks, regulation and differentiation of keratinocytes and their co-activators (Cabalín et al., 2021; Smith et al., 2021). Which in turn helps with growth regulation of keratinocytes in the human body (Lund et al., 2016; Claudel et al., 2019a). Autoflora sharing in the gastrointestinal tract affects individual gastrointestinal-skin axis, and in turn may indirectly affect the metabolism and availability of vitamins, such as Vitamin D, leading to changes the skin microbiome (Cogoli et al., 1984; Bikle et al., 2003; SanMiguel and Grice, 2015; Zwart and ScottSmith, 2020).

There is mounting evidence that some autoimmune pathologies are associated with low Vitamin D status, including the development of disordered skin growth (vitiligo and scleroderma) as well as heart disease, increased topical and systemic infection and diabetes development (Cogoli et al., 1984; Bikle, 2004; Bikle et al., 2004; DeLuca, 2004; Smith et al., 2009; Matheson et al., 2010; Hu et al., 2014; Zwart and ScottSmith, 2020). On a cellular level, lowered levels can affect murine and human plasmacytoid dendritic cellular function, creating a responsive-suppressive effect on cancer genesis and immune cell behavior (Zwart et al., 2013; Shen et al., 2014; Scott et al., 2021). Nasal colonisation by methicillin-resistant *Staphylococcus aureus,* MRSA*,* in individuals with low Vitamin D is recorded, demonstrating that commensals may out-compete other bacteria in a low Vitamin D environment (Penna, 2000; DeLuca, 2004; De Haes et al., 2005; Kostner et al., 2009; Smith et al., 2009; Matheson et al., 2010; Malodobra-Mazur et al., 2012; Karthaus et al., 2014; SanMiguel and Grice, 2015).

Future multi-generational long haul space flights may provide multiple mental health challenges, where mood management, cognitive stability and cooperative individuals will be needed for success. Vitamin D receptors are found in the brain, and a deficiency of Vitamin D can lead to decreases or alterations in cognitive functioning (Penna, 2000; Searing and Leung, 2010; Slominski et al., 2011; Pludowski et al., 2013; Saleh et al., 2013; Thill et al., 2015; Wierzbicka et al., 2015; Smith et al., 2021). Changes in the gastrointestinal-skin axis secondary to astronaut autoflora exchange may in turn impact Vitamin D production and the vitamin’s wider availability to the human immune system (Cogoli et al., 1984; Coiletti Louis et al., 1991; Taylor, 1993; Williams, 2003a; Bikle et al., 2003; Bikle, 2004; Bikle et al., 2004; Horneck and Comet, 2006; Kaur et al., 2008; Guéguinou et al., 2009; Carlson, 2014; Hu et al., 2014; Taylor, 2015; Zwart and ScottSmith, 2020; Spatz et al., 2021). Though supplementation guidelines are readily available, they may not be applicable in microgravity environments due to the acknowledged change in gastrointestinal microbiome and autoflora phenomena, creating dysfunction with the gastrointestinal-skin axis, complicated further by molecular structural stress (Adorini and Penna, 2008; Slominski et al., 2013d; Jennifer et al., 2021; Spatz et al., 2021). Oral supplementation presents itself as an obvious solution, but may also create challenges on longer haul space flights with regards to adequate supply (dosage for the protective and healthy immune response the human body requires) and changed gastrointestinal microbiome may lead to inadequate dosage uptake for individual needs (Taylor, 1974; Peterson et al., 2013; SanMiguel and Grice, 2015; NASA, 2021; Sole and Santamaria, 2021). UV supplementation is another avenue to explore, however Australian studies have shown that UV sun-damaged skin becomes compromised, falling behind on the production of Vitamin D utilized by other physiological systems in the body (Holick, 2004; Eckberg et al., 2005; Eyles et al., 2005; Heath and Elovic, 2006; Hawker et al., 2007; O’Hara and Shanahan, 2007; Garcia et al., 2011; Haussler et al., 2011; Duygu Gezen et al., 2012; Hu et al., 2014).

Extrapolating from bacteria and conformational molecular changes in microgravity, the warping of functional three dimensional (3D) structures (immunomolecular molecular structures) causes molecular structural stress, leading to further down regulation or dysfunctionality of keratinocyte co-activator contribution to kertinocyte growth and differentiation, creating an environment for mircobiome dysbiosis (Nickerson et al., 2000; Kaur et al., 2005; Kaur et al., 2008; Carlson, 2014; Picardo and Monica, 2014; Taylor, 2015; Schwager and Detmar, 2019; Jennifer et al., 2021; Spatz et al., 2021) In effect, along with compromised human immune functioning and wound healing, this can lead to longer and more infectious microorganism profiles (Schwager and Detmar, 2019; Cueto, 2022). Down-regulation of the human immune system in microgravity is competing with a range of pathogenic insults, including risk of encountering novel diseases in space, causing questions to be asked if humans could biologically survive in space (Nefedov et al., 1971; Guéguinou et al., 2009; Schwager and Detmar, 2019). It is becoming evident that general human health and well being is highly dependent on resident Vitamin D status (Smith et al., 1999; Holick, 2004; Wang et al., 2004; Eckberg et al., 2005; Eyles et al., 2005; Heath and Elovic, 2006; Hawker et al., 2007; Holick, 2007; O’Hara and Shanahan, 2007; Kaur et al., 2008; Misra et al., 2008; Garcia et al., 2011; Haussler et al., 2011; Zwart et al., 2011; Bikle, 2012; Duygu Gezen et al., 2012; Slominski et al., 2013d; Hossein-nezhad and Holick, 2013; Zwart et al., 2013; Carlson, 2014; Zwart and ScottSmith, 2020; Scott et al., 2021; Smith et al., 2021; Cueto, 2022).

## Microbiological and viral behaviour and pharmacological resistance in microgravity

Many potential pathogens inhabit and colonise the human body, and apart from a possible direct pathogenic threat, gastrointestinal commensal balance can also affect the absorption and activation of much needed vitamins and nutrients to maintain a healthy functioning immune system (Canova Sabrina et al., 2005; Carlson, 2014; Wierzbicka et al., 2014). Space flights have been demonstrated to cause changes to the gastrointestinal and gastrointestinal-skin axis and nasal colonisation with resident respiratory bacterial flora (Taylor, 1993; Stowe et al., 2001; Du et al., 2002; Baqai et al., 2009; Guéguinou et al., 2009; Du et al., 2011; Taylor, 2015; Smith et al., 2021; Sole and Santamaria, 2021; Spatz et al., 2021; Tang et al., 2021). Maintaining a healthy microbiome is challenged by a two-fold antagonistic process**:** the rapid growth of potential pathological organisms (up to four times Earth-bound growth rates), coupled with the noted lack of medication stability and effectiveness in space (often discussed as increased resistance) (Du et al., 2002; Malodobra-Mazur et al., 2012; Taylor et al., 2012; Wierzbicka et al., 2014; Taylor, 2015; Jennifer et al., 2021; Spatz et al., 2021). Anaerobic and aerobic organisms experience decreased generational replication times, perhaps not allowing uptake of systemic antibiotic agents at the correct replication phase for effectiveness, potentially rendering antibiotic treatments minimally effective.

Additionally, changes in the nature and type of cytoskeleton expression appear to provide some species of bacteria a competitive advantage. *Salmonella typhimurium*, *Staphylococcus epidermis* (a normal skin commensal) have demonstrated predatory behavior with these changes, effectively out-completing other commensals (Nefedov et al., 1971; Coiletti Louis et al., 1991; Nickerson et al., 2000; Du et al., 2002; Picardo and Monica, 2014; Taylor, 2015; Lund et al., 2016; Jennifer et al., 2021). Further research is investigating the effects of radiation and microbiological organisms genetic response (Wang et al., 2004). Genetic mutations have been noted in *Saccharomyces cerevisiae* after longer space flights, and changed *Escherichia coli* gene expression is noted. These changes, coupled with host human epigenetic ageing (in response to radiation, stress, and microgravity environment) provide a compromised course for the senescence immune response (Sonnenfield and Taylor, 1991; Klaus et al., 1997; Fukuda et al., 2000; Wang et al., 2004; Karthaus et al., 2014; Nickerson et al., 2016; Zea et al., 2017; Nwanaji-Enwerem Jamaji et al., 2020).

Some microorganisms have demonstrated persistent resident populations in microgravity, surviving successive space flights (Aunins Thomas et al., 2018; Singh et al., 2018; More et al., 2019; Mortazavi, 2019). These include those indicated as a biosafety level 2 microorganism: *Acinetobacter baumannii*, *Haemophilus influenzae*, *Klebsiella pneumoniae*, *Salmonella enterica*, *Shigella sonnei*, *Staphylococcus aureus*, *Yersinia frederiksenii*, and *Aspergillus lentulus* (Aunins Thomas et al., 2018). Of the persistent space flight bacteria, *Staphylococcus aureus and Yersinia frederiksenii* are known skin pathogens, and *Aspergillus lentulus, Haemophilus influenzae*, and *Klebsiella pneumoniae* are more routinely encountered clinically in the human respiratory system. Whilst *Salmonella enterica* and *Shigella sonnei* are mainly considered gastrointestinal pathogens, *Acinetobacter baumannii* has demonstrated its pathological capabilities*,* particularly with indwelling urinary catheters in the immunocompromised individuals experiencing prolonged hospitalization (Bijlani et al., 2021). Although the conditions in space habitats and microgravity environments are selective, they do not alter the micro-biological affect on human health and the same disease profile can be expected with a predicted quicker timeline (Singh et al., 2018; More et al., 2019; Mortazavi, 2019).

Resistance *per se* may not be the correct conceptual terminology to describe the ineffectiveness of antibiotics, for example resistance may be due to faster generational/replication time, affecting antibiotics and antiretrovirals *introduction* into the organisms at the “right moment”to disrupt their replication pathways. Given the expected change in gut flora due to autoflora colonisation in astronauts, storage and antibiotic resistance concerns, the bio-availability of therapeutics in long space flight will need to be considered (Taylor, 1974; Teize and Putcha, 1994; Du et al., 2002; Canova Sabrina et al., 2005; Du et al., 2011; Putcha et al., 2011; Taylor et al., 2012; Turroni et al., 2020). Medication storage in microgravity and the effect of increased radiation (over periods of time) coupled with other (as yet) unquantifiable factors, may well impact on medication integrity (Nefedov et al., 1971; Coiletti Louis et al., 1991; Smith et al., 1999; Nickerson et al., 2000; Stowe et al., 2001; Du et al., 2002; Williams, 2003a; Canova Sabrina et al., 2005; Kaur et al., 2005; Kaur et al., 2008; Baqai et al., 2009; Guéguinou et al., 2009; McPhee and Charles, 2010; Du et al., 2011; Zwart et al., 2011; Zwart et al., 2013; Carlson, 2014; Mehta et al., 2014; Picardo and Monica, 2014; SanMiguel and Grice, 2015; Schwager and Detmar, 2019; Cabalín et al., 2021; Jennifer et al., 2021; Solari et al., 2021; Spatz et al., 2021). As treatment options become compromised, and antibiotics lose a level of their effectiveness, resistance will inevitably increase, particularly with rapid generational rates (Taylor et al., 2012). It is evident the nature of long haul space flight will create the conditions conducive to dysbiosis of the skin microbiome, autoflora changes will impact Vitamin D production, which in turn affects skin barrier growth and function, encouraging barrier dysfunction (Du et al., 2011).

## The effects of autoflora exchange and skin changed pH

Each individual provides an ecosystem of variable habitats, with bacterial, viral and fungal commensals demonstrating preferential environments. Colonization and autoflora exchange in closed environments is a known effect of both simulated and actual space environments; individual physiology dictates how a population of flora will inhabit different individuals. Gastrointestinal autoflora exchange has been quite well documented (Zea et al., 2017; Aunins Thomas et al., 2018; Singh et al., 2018; Mortazavi, 2019). Conversely, the mechanisms behind skin autoflora exchange and microbiological persistence in space flight vehicles and analogs is not well understood (Taylor and Sommer, 2005; Maloney et al., 2014; Aunins Thomas et al., 2018; Turroni et al., 2020; Yu et al., 2020). The microbiome of the gut and skin is now understood to be shared between participants who are housed in closed quarters (Williams, 2003b; Kostner et al., 2009; Karthaus et al., 2014; Mann et al., 2019). Skin commensal exchange, even when aseptic techniques and sterility is maintained, is a little more difficult to catalog. One presumes that the hygiene is strong in space vehicles and analog environments (Naik et al., 2012; Voorhies and Lorenzi, 2016; Tan et al., 2017; Ehrhardt, 2018; Rainer Barbara et al., 2020; NASA, 2021). The persistence and increased virulence of some microbiological organisms in space vehicles would suggest a more subtle transference vector (Zea et al., 2017; Aunins Thomas et al., 2018; Surber et al., 2018b; Mann et al., 2019).

As outlined, multivariate factors impact on skin health, and the relatively recent understanding of these factors working together to create a healthy skin environment has highlighted the fragility of the system once outside the Earthly environment (Horneck and Comet, 2006; Peterson et al., 2013). It is recognised that skin immunity is contributed to by resident commensals, and skin health can be affected by a change in the population dynamics of the resident commensals, and a change in the surface pH (NASA, 2021; Sole and Santamaria, 2021). Earth-bound studies have shown that pathological conditions such as rosacea and acne can be communicable, with individuals demonstrating a genetic propensity for these diseases which thrive in an alkaline pH environment [(Naik et al., 2012; Hu et al., 2014; Voorhies and Lorenzi, 2016; Tan et al., 2017; Surber et al., 2018a; Ehrhardt, 2018; Rainer Barbara et al., 2020), 135].

Changes in pH can greatly affect the habitat and composition of skin microbiome (Wheatcroft, 1989; Naik et al., 2012; Angelova-Fischer et al., 2018; Kahraman et al., 2019; Farkas and Farkas, 2021). This in turn, when coupled with human immune system dysfunction, could be expect to lead to disease proliferation, i.e., latent common viral diseases reactivating in microgravity environments, and novel and persistent habitat infiltration (Taylor and Sommer, 2005; Maloney et al., 2014; Turroni et al., 2020; Yu et al., 2020). Novel technical solutions for maintaining or repopulation of a healthy microbiome may include autoflora generalisation (generic autoflora distribution across individuals in a closed environment before long-haul space flight) of gastrointestinal and skin microbiome flora to maximise vitamin and nutrient absorption (Williams, 2003a).

## Skin health, wound healing, and optimal pH

A survey of reported medical issues during space flights has demonstrated that dermatological issues appear as the most regular complaint (Stowe et al., 2001; Horneck and Comet, 2006; Mehta et al., 2014). Skin complaints after space travel have included dryness, eczema, itch, acne and other infections, thinning skin, and erythemous or mastoidcyotic reactions to stimuli, suggesting changes to the barrier functioning of the skin and creation of dybiosis (Wheatcroft, 1989; Naik et al., 2012; Youn et al., 2013; Angelova-Fischer et al., 2018; Surber et al., 2018b; Kahraman et al., 2019; Farkas and Farkas, 2021). These factors can profoundly impact astronaut health, well-being and comfort in microgravity. Skin dysbiosis can act as a medical distraction, and should be a cardinal consideration on long haul space flights (Williams, 2003a). It is further appreciated that both short and long duration space flight have the potential to severely disrupt the skin microbiome, which will also be prone to currently unquantifiable radiation influences (Taylor, 1974; Penna, 2000; DeLuca, 2004; De Haes et al., 2005; O’Hara and Shanahan, 2007; Adorini and Penna, 2008; Kostner et al., 2009; Smith et al., 2009; Matheson et al., 2010; Searing and Leung, 2010; Slominski et al., 2011; Malodobra-Mazur et al., 2012; Slominski et al., 2013d; Peterson et al., 2013; Pludowski et al., 2013; Saleh et al., 2013; Hu et al., 2014; Karthaus et al., 2014; Thill et al., 2015; Wierzbicka et al., 2015; NASA, 2021; Sole and Santamaria, 2021).

Optimal skin health occurs within a range of pH value. Depending on the location/site of the human body, a number of factors affect skin pH: age, anatomical site, genetic and ethic inheritance, sebum, sweat and skin moisture (Wheatcroft, 1989; Naik et al., 2012; Angelova-Fischer et al., 2018; Kahraman et al., 2019; Farkas and Farkas, 2021). As an example of ethic inheritance affecting skin behaviour, with darkly pigmented skin demonstrates a lower pH and far superior barrier integrity than lighter skin (Naik et al., 2012; Farkas and Farkas, 2021). The pH tends to attract and help stabilise the human microbiome ecosystem within the realms of its symbiotic relationship, and the microbiome is greatly affected by pH and changes in pH (Wheatcroft, 1989). Newly hypothesized dysbiosis causative agents, such as *Staphylococcus epidermidis*, persist in the microgravity environment (Wheatcroft, 1989; Naik et al., 2012; Youn et al., 2013; Surber et al., 2018b; Farkas and Farkas, 2021).

Bacteria that comprise the microbiome contribute growth factors and peptides to the skin, stimulating skin repair. *Staphylococcus aureus* is one of the mainstays of this contribution, but may be outcompeted by *Staphylococcus epidermidis*, having an additional deleterious effect on wound healing (Youn et al., 2013; Surber et al., 2018a; Surber et al., 2018b). Common pathogenic bacteria such as *Staphylococcus aureus* show optimal growth at pH of 7.5, whilst *Propionibacterium acnes* has optimal growth at pH 6.3, and the newer organism contributing to acne, the bacteria *Staphylococcus epidermidis* is persistent in space flight, also preferring a more alkaline pH (Jürgen et al., 2011; Voorhies and Lorenzi, 2016; Tan et al., 2017; Surber et al., 2018a; Ehrhardt, 2018; Nanna, 2018; Claudel et al., 2019b; Kahraman et al., 2019; Lynde et al., 2019; Mortazavi, 2019; Cueto, 2022). The hydrophobic character and lipid distribution of the stratum corneum requires the organisation of lipids into a series of lamellar layers, and the synthesis of ceramides greatly improves with a lowered pH (Naik et al., 2012). It has been demonstrated that a lowered pH improves barrier functioning and integrity by creating an environment whereby ceramides can be generated by maximal enzymatic reaction of critical components of the permeability barrier of the skin. Two important enzymes, *β*-glucocerebrosidase and acidic sphingomyelinase, work at specific pH of 5.6 and 4.5 respectively (Naik et al., 2012; Angelova-Fischer et al., 2018; Kahraman et al., 2019). The change to sebum composition, which occurs under hormonal influences, reduces fatty acids presence and increases squalene and pH.

The basic principles for management of skin microbiome should include correcting pH, a balanced level of fatty acids present (secreted by the resident flora), and avoiding substances that damage or disrupt the microbiome. Treatment of elderly skin, which suffers from much of the same complaints as those astronauts who have experienced space flight, also suggest appropriate lowering of the pH in skincare products (Youn et al., 2013; Claudel et al., 2019b). Impaired wound healing has been documented in the space environment. Factors such as 3D molecular structural stress, changes in pH and barrier function, may lead to compromised functioning and contributing to noted skin atrophy and poor skin health of returning astronauts (Youn et al., 2013; Schreml et al., 2014; Neutelings et al., 2015; Braun et al., 2019; Lynde et al., 2019; Afshinnekoo et al., 2020; Cubo-Mateo and Gelinsky, 2021). Newer techniques, being developed on Earth (stem cell culture, bio-mechanical printing, placental growth factor harvesting, patches to deliver growth factors and progenitor wound healing molecules), may not have the availability or same behavior in the microgravity environment to be of value in space flight (Skardal et al., 2012; Neutelings et al., 2015; Widgerow et al., 2016; Dyer and Miller, 2018; Braun et al., 2019; Tottoli et al., 2020). Medications and treatments for infection, skin health maintenance and pH balance will face manufacturing, medication stability, and storage challenges in the remote and potentially hostile environment of spacecraft designed for long-duration space flight. A lowered skin pH would increase the wound healing capabilities of the skin, and could be as simple as using slightly acidic wipes for washing (without removal), to promote an acidic environment, perhaps three times a week whilst in the microgravity environment.

## Discussion

In the overall concext of space travel, it is becoming obvious that the area of medicine and astronaut health which may become cardinal to ensuring successful missions is maintenance of a healthy skin microbiome within the microgravity environment (Horneck and Comet, 2006; Stewart et al., 2007; Taddeo et al., 2008; Skardal et al., 2012; Saba, 2013; Tottoli et al., 2020; Paul et al., 2021). It is now appreciated that space flight contributes to skin dysbiosis, including, but not limited to, dryness, acne, topical infections, (with recorded increased virulence/reactivation of a herpetic/viral infections) and slowed wound healing.

This admix of poor barrier function, compromised immune response, the impact of changed individual autoflora, and disruptions of the gastrointestinal-skin axis (i.e. impaired Vitamin D metabolism and deficiency), together with poor wound healing, has the potential to lead to skin dysbiosis. The collective storm of decreased/altered human immune response, decreased wound healing, changes to microbiome, coupled with biologically demonstrated increased microbe replication and infective states (with reduced antibiotic and antiretroviral effectiveness), will inevitably lead to increased dysbiosis and risk of infections. Microgravity-induced 3D molecular stress, leading to molecular dysfunctionality of co-enzymes and interruptions of signalling/immune cellular recruitment pathways, cannot be ignored in planning for long-duration space flight. Consideration of innate pharmaceutical failure (packaging, longevity of medications, radiation damage), and ability to manufacture specifically designed medications, coupled with packaging and preservation, it is essential to give good “shelf life” for vital medicinal, food and personal care products during long-duration spaceflights. By definition, in normal use, sealed packaging is designed to prevent microbial growth, and thus is a limiting factor for microbiome-restorative medicaments.

Ensuring optimal functioning of the skin microbiome is paramount, but what are some practical measures to ensure this happens? The sebum consists of fatty acids, which are liberated by the microbiome, and are one of the mechanisms by which the skin remains acidic. It is acknowledged that an acidic pH is vitally important to the microbiome health, and in turn the biomechanics of the underlying human tissue (Naik et al., 2012; Youn et al., 2013; Angelova-Fischer et al., 2018; Kahraman et al., 2019; Lynde et al., 2019). A logical scenario would be to replicate the environment in which we know the microbiome exists optimally, bar the effects of microgravity. Whilst still in its infancy, there is enough data to piece together evidence-based strategies on such a concept. It is now known that in higher or more alkaline pH the microflora of the skin changes its composition and certain bacterial and viral species become more virulent (Jürgen et al., 2011; Nanna, 2018; Kahraman et al., 2019; Rainer Barbara et al., 2020; Farkas and Farkas, 2021). New data suggests that a lower than expected pH would create a healthier environment for skin’s microflora (Stewart et al., 2007; Taddeo et al., 2008; Saba, 2013; Youn et al., 2013; Eshelby, 2021; Paul et al., 2021; Cueto, 2022). One aspect of skin health in the changed immunological environment of microgravity, the pH, could be a simple, but effective, way to increase skin health, decrease topical infection rate, increase skin immune responses, and maintain a healthy biome (Youn et al., 2013; Lynde et al., 2019).

The authors suggest consistently maintaining a lowered skin pH in the microgravity environment as a direct methodology for maintaining astronaut skin barrier health whilst reducing the risk of infection. This approach would result in the maintenance of the skin barrier, reduce dryness, and slow disease development and topical infections (such as acne and rosacea), whilst helping at the same time to maintain the skin’s microbiome (Jürgen et al., 2011; Youn et al., 2013; Nanna, 2018; Lynde et al., 2019). A lowered pH would lead to increased skin wound healing capabilities.

## Conclusion

International space agencies are planning for long-duration human spaceflight. However at our current level of technology, they present several medical challenges for human health. Good health and wellbeing for spacefarers has become a complex immunological, nutritional and medical issue, whereby new technology and solutions for expected and novel diseases and infections require careful thought and deployment of resources. Keeping crews fit and healthy (accounting for age-related disease profiles) require consideration of the biological, physiological, and pathogenic changes stemming from both potential pathogens, changes to skin microbiome, gastrointestinal autoflora, and the gastrointestinal-skin axis. It is also important to account for the molecular structural stress experienced in microgravity environment, and the effect of this on the human immune system.

Reducing skin pH, adequate Vitamin D supplementation, maintenance of the gastrointestinal-skin axis, and protection and monitoring of the skin microbiome, may provide a novel prophylactic and treatment course to help maintain a healthy individual skin microbiome. Improving and maintaining skin microbiome quality will reduce the risk of skin dysbiosis and infections during long-haul space flights.

## Data Availability

The original contributions presented in the study are included in the article/supplementary material, further inquiries can be directed to the corresponding author.
